# Constipation, deficit in colon contractions and alpha-synuclein inclusions within the colon precede motor abnormalities and neurodegeneration in the central nervous system in a mouse model of alpha-synucleinopathy

**DOI:** 10.1186/s40035-019-0146-z

**Published:** 2019-02-06

**Authors:** Lucia Rota, Carolina Pellegrini, Laura Benvenuti, Luca Antonioli, Matteo Fornai, Corrado Blandizzi, Antonino Cattaneo, Emanuela Colla

**Affiliations:** 1 Bio@SNS Laboratory, Scuola Normale Superiore, Piazza dei Cavalieri 7, 56126 Pisa, Italy; 2 Department of Pharmacy, University of Pisa, Via Roma 55, 56126 Pisa, Italy; 3 Department of Clinical and Experimental Medicine, University of Pisa, Via Roma 55, 56126 Pisa, Italy; 4 Neurotrophins and Neurodegenerative Diseases Laboratory, Rita Levi-Montalcini European Brain Research Institute, Viale Regina Elena 295, Rome, 00161 Italy

**Keywords:** Alpha-synuclein, Constipation, Gastrointestinal dysfunction, Bowel dysmotility, Non-motor symptoms, Parkinson’s disease, Enteric nervous system, Alpha-synucleinopathy

## Abstract

**Background:**

Gastrointestinal dysfunction can affect Parkinson’s disease (PD) patients long before the onset of motor symptoms. However, little is known about the relationship between gastrointestinal abnormalities and the development of PD. Contrary to other animal models, the human A53T alpha-synuclein (αS) transgenic mice, Line G2–3, develops αS-driven neurological and motor impairments after 9 months of age, displaying a long presymptomatic phase free of central nervous system (CNS) dysfunction.

**Methods:**

To determine whether this line can be suitable to study constipation as it occurs in prodromal PD, gastrointestinal functionality was assessed in young mice through a multidisciplinary approach, based on behavioral and biochemical analysis combined with electrophysiological recordings of mouse intestinal preparations.

**Results:**

We found that the A53T αS mice display remarkable signs of gastrointestinal dysfunction that precede motor abnormalities and αS pathology in the CNS by at least 6 months. Young αS mice show a drastic delay in food transit along the gastrointestinal tract, of almost 2 h in 3 months old mice that increased to more than 3 h at 6 months. Such impairment was associated with abnormal formation of stools that resulted in less abundant but longer pellets excreted, suggesting a deficit in the intestinal peristalsis. In agreement with this, electrically evoked contractions of the colon, but not of the ileum, showed a reduced motor response in both longitudinal and circular muscle layers in αS mice already at 3 months of age, that was mainly due to an impaired cholinergic transmission of the underlying enteric nervous system. Interestingly, the presence of insoluble and aggregated αS was found in enteric neurons in both myenteric and submucosal plexi only in the colon of 3 months old αS mice, but not in the small intestine, and exacerbated with age, mimicking the increase in transit delay and the contraction deficit showed by behavioral and electrical recordings data.

**Conclusions:**

Gastrointestinal dysfunction in A53T αS mice represents an early sign of αS-driven pathology without concomitant CNS involvement. We believe that this model can be very useful to study disease-modifying strategies that could extend the prodromal phase of PD and halt αS pathology from reaching the brain.

**Electronic supplementary material:**

The online version of this article (10.1186/s40035-019-0146-z) contains supplementary material, which is available to authorized users.

## Background

Along with typical motor dysfunctions, Parkinson's disease (PD) patients experience a variety of non-motor symptoms that involve both the central nervous system (CNS) and the peripheral nervous system (PNS), with a deep impact on their quality of life. Among them, constipation and anosmia can manifest even decades before the onset of the motor abnormalities, and thus their analysis can be potentially useful for early diagnosis and therapeutic intervention [1]. Constipated patients display infrequent bowel movements, impairment of propulsive colonic motility, prolonged colonic transit time, reduced rectal contractions and abnormalities in motor activity of the anal sphincter [2]. Affecting up to 80% of PD patients, constipation represents the most frequent gastrointestinal (GI) dysfunction in PD [3] and patients with a previous diagnosis of constipation have an increased risk of developing PD [4], e.g. less than 1 bowel movement per day is linked to a 2.7-fold increase in risk of PD [5]. However, very little is known about the relationship between bowel dysfunctions and development of PD.

The pathological hallmarks of PD, Lewy bodies (LBs) and Lewy neurites, abnormal proteinaceous inclusions mainly composed of alpha-synuclein (αS) present in the brain of the patients [6], have been also found in the enteric nervous system (ENS) [7–10], which is the network responsible for the whole innervation of the gut. In addition, several studies have implicated αS neuronal transmission and propagation within the nervous system as a mechanism of detrimental spreading of PD pathology, showing that neuron-to-neuron transfer of pathogenic αS occurs both in vitro [11–13] and in animal models [14–20] and suggesting that the accumulation of toxic αS species may originate outside the CNS and later move to the brain using anatomical connections [21, 22]. In line with these findings, detection of αS inclusions in the large intestine of human subjects has been investigated as a promising diagnostic tool in living patients, as samples can be easily obtained through biopsies [23]. However, because of the presence of enteric phosphorylated αS aggregates in healthy controls [24, 25], variable specificity of αS antibodies employed [7, 8] and the heterogeneity in the intestinal areas analysed [26], the detection of αS inclusions in colonic biopsies as a reliable PD biomarker is still highly controversial [27, 28].

On the other hand, in terms of animal models, until now, there has been a lack of a suitable paradigm that would allow the study of constipation as it occurs in the prodromal phase of PD, without overt motor dysfunction. Indeed, the vast majority of current genetic or chemically induced models present GI deficits and αS accumulation in the gut concomitant with impaired motor activity or αS pathology in the CNS [29–33], making it difficult to understand the progressive nature of constipation during the PD prodromal phase and how it relates to CNS neuropathology. Here, we show that the PrP human A53T αS transgenic (Tg) mice, line G2–3, [34], one of the first genetic model developed to study α-synucleinopathy, displays GI dysfunction with simultaneous accumulation of αS inclusions in the ENS, months before the typical onset of motor abnormalities, neuronal degeneration and formation of αS positive inclusions in the CNS. In this model, constipation represents an early sign of αS-driven pathology that is temporally unrelated to CNS involvement. Because of this, we believe that the A53T αS G2–3 line can be particularly useful to study the contribution and the influence of constipation on αS-driven CNS neuropathology.

## Methods

### Mice

Tg mice expressing human A53T αS under the control of the mouse prion protein (PrP) promoter [line G2–3 (A53T)] have been described previously [34, 35]. This model develops neurological abnormalities as early as 9 months of age (with an average peak at ~ 13 months) that manifest initially with reduced locomotion, wobbling, lack of balance and weakness of the hind limbs. This disease phenotype becomes progressively more severe, culminating in a fatal paralysis within 14–21 days from the onset of the first symptoms. Because of the high variability in time of onset (between 9 to 16 months), Tg mice are closely monitored after 9 months of age with the understanding that once the first symptoms appear, that mouse is committed to develop the full phenotype. Diseased mice show an accumulation of intracellular, phosphorylated (serine 129) and ubiquitinated αS inclusions, neuroinflammation and neuronal degeneration in CNS [34]. For the purpose of this study, sick Tg mice at 12–14 months, presymptomatic mice at 3, 6, 9 and 12 (if still healthy) months, and age-matched nTg littermate controls were used. All animal studies were approved by and complied in full with the national and international laws for laboratory animal welfare and experimentation (EEC council directive 86/609, 12 December 1987 and Directive 2010/63/EU, 22 September 2010).

### Behavioral experiments

Behavioral analysis was carried out on groups of 20–30 mice, whereas for the gait test, food intake and glucose blood level groups consisted of ~ 10 mice each. Both male and female animals were used. Each trial was performed 1 to 3 times per animal on non-consecutive days.

#### Gait test

The gait test was assessed in mice after o/n starvation. Each animal’s paw was painted with blue washable paint and the mouse was allowed to walk onto a white paper strip, at the end of which a piece of mouse chow was placed as a food reward. The footprints were circled and allowed to dry. Stance length, sway distance and stride length were measured for each mouse.

#### Whole gut transit time

Whole gut transit time (WGTT) was assessed in mice after oral gavage of a 0.05 mL chocolate milk containing 4% of Brilliant Blue food dye. Post-gavage, the animals were observed until the time of excretion of the first blue stool, which was recorded for each mouse.

#### Stool collection

Stool collection assays were performed between 9:00 AM and 11:00 AM on each day. Each animal was removed from its home cage and placed in a clean plastic cage without food or water for 1 h. Stools were collected immediately after expulsion and placed in sealed tubes. At the end of the trial, the stools were counted and weighed (total weight), then dried o/n at 65 °C and weighed again to provide dry weight. Water content was calculated as a difference between total weight and dry weight.

#### Food intake

Each animal was removed from its home cage and single-housed for 24 h with free access to food and water. Food was weighed before and after the trial and food intake was calculated as a difference between the two amounts.

#### Glycemia

Glucose levels were assessed using reactive stripes (OneTouch Verio, LifeScan Italia, Milan, Italy) with a single drop sample of blood taken from the tail of each animal. Glucose level was analyzed after o/n food removal and after the mice were allowed free access to chow for 1 h.

### Recording of contractile activity from longitudinal and circular muscle preparations of colon and ileum

Contractile activity of colonic or ileal longitudinal and circular smooth muscle was recorded as previously described [36, 37]. After sacrifice, the colon and the ileum were removed and placed in cold Krebs solution. Longitudinal and circular muscle strips of the intestine were set up in organ baths containing Krebs solution at 37 °C, bubbled with 95% O_2_ + 5% CO_2_. The strips were connected to isometric force transducers (2Biological Instruments, Besozzo, Italy). Mechanical activity was recorded as a measure of tension using a BIOPAC MP150 system (2Biological Instruments). A pair of coaxial platinum electrodes was positioned at a distance of 10 mm from the longitudinal axis of each preparation to deliver transmural electrical stimulation (ES) by a BM-ST6 stimulator (Biomedica Mangoni, Pisa, Italy). ES were applied as 10-s single trains consisting of square wave pulses (0.5 ms, 30 mA). To measure muscle contractility of the colon or the ileum, electrically evoked motor responses were recorded from tissue preparations maintained in standard Krebs solution. To measure the neurogenic contribution to muscle contraction, electrically evoked motor responses were recorded after selective stimulation of the nitrergic, cholinergic or NK1-mediated tachykinergic pathways from colonic preparations maintained in Krebs solution containing respectively: guanethidine (10 μM), L-732,138 (10 μM), GR159897 (1 μM), SB218795 (1 μM) and atropine (1 μM) in order to inhibit the noradrenergic, NK-mediated tachykinergic and cholinergic pathways, while recording the nitrergic signal; L-NAME (100 μM), guanethidine (10 μM), GR159897 (1 μM), SB218795 (1 μM) and atropine (1 μM) in order to prevent the recruitment of the nitrergic, noradrenergic, NK2 and NK3-mediated tachykinergic and cholinergic systems, while recording the NK1-mediated tachykinergic pathway; L-NAME, guanethidine, L-732,138, GR159897, SB218795, in order to record the cholinergic response while inhibiting the nitrergic, noradrenergic and tachykinergic signals. To evaluate the myogenic contribution to the total contractile activity of the colon, muscle response was evoked by direct pharmacological activation of muscarinic receptors located on smooth muscle cells. For this purpose, colonic preparations were maintained in Krebs solution containing tetrodotoxin (1 μM) and stimulated with carbachol (10 μM). The tension developed by each preparation (grams) was normalized by the wet tissue weight (g/g tissue). All the chemical compounds were purchased from Sigma Aldrich (St. Louis, Missouri USA).

### Tissue collection and western blot analysis

For biochemical analysis the mouse intestine was cut into 6 segments corresponding to duodenum (D), jejunum (J), proximal ileum (PI), distal ileum (DI), proximal colon (PC) and distal colon (DC), flushed of fecal contents, opened longitudinally, scraped for removing the epithelium and minced. Cold phosphate-buffered saline (PBS), with proteases and phosphatases inhibitors, was used for this procedure. All samples were frozen on dry ice immediately after collection and stored at − 80 °C until use. Frozen intestine segments were homogenized using a Potter-Elvehjem Grinder homogenizer on ice in 20% (*w*/*v*) TNE lysis buffer (50 mM Tris-HCl pH 7.4, 100 mM NaCl, 0.1 mM EDTA) with proteases and phosphatases inhibitors. An equal volume of TNE buffer containing 2% of NP-40 was added to initial homogenates that were then centrifuged at 10,000 x *g* at 4 °C in order to collect NP-40 soluble and insoluble fractions. Pellets were then washed one time with TNE buffer with 1% of NP-40 and resuspended in 10% of the original homogenization volume in TNE containing 1% NP-40, 1% SDS, 0.1% DOC. NP-40 insoluble fractions were then sonicated and boiled for 5 min at 95 °C. Protein amount was determined with BCA. Immunoblot analyses were performed as previously described [13]. Briefly, lysates were run on a 4–20% Criterion™ TGX™ Precast Midi Protein Gel (Bio-Rad, Hercules, CA, USA) and then transferred onto nitrocellulose membrane at 200 mA, o/n at 4 °C, using carbonate transfer buffer (10 mM NaHCO_3_, 3 mM Na_2_CO_3_, 20% MeOH). Transfer efficiency was controlled by Ponceau staining. Unspecific binding sites were blocked by 30 min membranes incubation with 5% nonfat dry milk (Bio-Rad) in 1X PBS containing 0.01% Tween-20 (PBS-T) at RT. Membranes were then incubated with the specific primary antibody dissolved in 2.5% nonfat dry milk in PBS-T, o/n at 4 °C. The following primary antibodies were used: Syn-1 (BD Biosciences, NJ, USA), pser129-αS (EP1536Y, Abcam), GAPDH (Thermofisher, Carlsbad, CA, USA), α-Tubulin (Sigma-Aldrich). Membranes were washed with PBS-T and incubated for 1 h at RT with the appropriate horseradish peroxidase-conjugated secondary antibody in 2.5% nonfat milk in PBS-T. Only for pser129-αS detection, 1X Tris-buffered saline containing 0.01% Tween-20 instead of PBS-T was used for the entire procedure. The chemiluminescent signals were visualized using a CCD-based Bio-Rad Molecular Imager ChemiDoc System. Band intensities were analyzed using Quantity One software (Bio-Rad).

### Immunofluorescence analysis

For immunofluorescence analysis, mice were perfused with 4% paraformaldehyde (PFA) in PBS 1X after 1 mL intraperitoneal injection of 2% *w*/*v* Tribromoethanol. The colon was collected and flushed of fecal contents, post-fixed o/n in 4% PFA/PBS at 4 °C and stored in maintenance solution (30% sucrose, 0.1% NaN_2_ in PBS) at 4 °C. A segment of 2 cm of distal colon was embedded in Tissue-Tek® OCT (Sakura, The Netherlands), cut at the cryostat in serial 12 μm sections and mounted on SuperFrost Plus glass slide (Thermofisher). Slides were then washed with PBS and let dry for ~ 3 h at 37 °C. Sections were then incubated with blocking solution [3.5% fat dry milk, 0.3% Triton X-100 (Tx-100), 6% normal goat serum (NGS) in PBS] for 1 h at RT and then incubated with primary antibody o/n at RT in blocking buffer. The following antibodies were used: pser129-αS and LB509 (Abcam), β-3-Tubulin and Syn204 (Cell Signaling Technology, Danvers, MA, USA), ChAT and Tyrosine Hydroxylase (TH) (Millipore, Burlington, MA, USA). On the next day, the sections were washed twice in PBS and incubated with Alexa Fluor secondary antibodies (ThermoFisher) in PBS containing 1.5% NGS, 0.3% Tx-100 for 1 h at RT. Sections were counterstained with Dapi and mounted on a glass slide using Fluormount (Sigma-Aldrich).

### Statistical analysis

All values are expressed as the mean ± S.E.M. Differences between means were evaluated by two-way ANOVA, followed by Fisher LSD post-hoc test (Prism, Graph Pad Software, San Diego, CA).

## Results

### Constipation in Prp A53T αS Tg mice is already present at 3 months of age in absence of overt motor dysfunction and accumulation of αS CNS inclusions

**Fig. 1:**
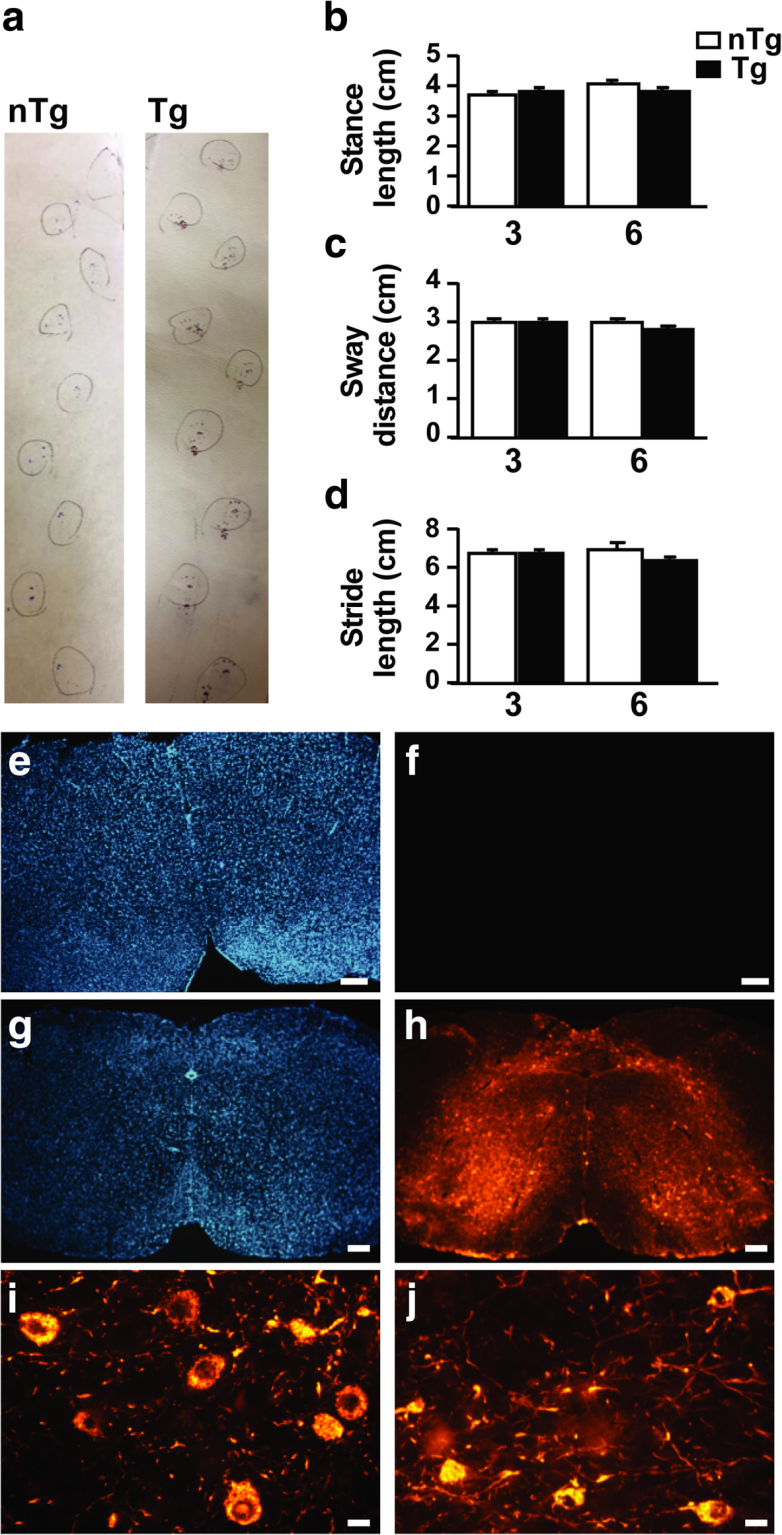
αS Tg presymptomatic mice do not display motor deficits nor αS pathology in the CNS. Young presymptomatic αS Tg at 3 and 6 months were evaluated for αS-driven pathology and motor abnormalities. During the gait test mice were allowed to walk on a white paper strip with painted paws and stance length, sway distance and stride length were measured. The walking pattern in 3 and 6 months old Tg and nTg mice remained unchanged. **a** Representative walking pattern of presymptomatic 6 months Tgs and nTg littermate do not show differences in stride and stance length and sway distance. **b**, **c**, **d** Graphs of gait parameters analyzed in 3 and 6 months old mice. Values on graphs are expressed as raw data and are given as the mean ± SEM (*n* = 5/10 per group), two-way ANOVA followed by Fischer’s LSD test. **e**, **f**, **g**, **h**, **i**, **j** Immunofluorescence analysis of pons in presymptomatic (9 months) (**e**, **f**) or sick (**g**, **h**, **i**, **j**) αS Tg mice stained with pser129-αS antibody (**f**, **h**, **I**, **j**) or DAPI (**e**, **g**). Phosphorylated pathological αS was found only in sick Tg mice after onset of neurodegeneration, indicating the lack of αS-driven pathology in the CNS of adult presymptomatic animals. Images were acquired with Nikon epifluorescence microscope, objective 4x, scale bar = 100 μm (**e**, **f**, **g**, **h**) or objective 40x, scale bar = 30 μm (**i**, **j**)

To determine if Prp A53T αS Tg mice can be used as a suitable model to study constipation as it occurs in the prodromal phase of PD, we first analyzed motor deficit and correlated accumulation of αS-positive inclusions in the CNS, two typical features of this model, in young and adult presymptomatic mice, to exclude that subtle changes in the αS-driven phenotype were already present at a young age before the appearance of full-blown motor dysfunction. In the presymptomatic stage these mice did not show any gross motor abnormalities, maintained a normal gait (Fig. 1a, b) and balance [34] while no presence of phosphorylated (serine 129) αS inclusions in the midbrain (Fig. 1g) and spinal cord [13] of Tg animals at 9 months was found, confirming that the appearance of αS-driven pathology in the CNS is closely linked to and concomitant with the onset of motor dysfunction and neurodegeneration. Additionally, presymptomatic mice up to 9 months of age did not show signs of neuronal dysfunction in the CNS including the appearance of endoplasmic reticulum (ER) stress markers, ER stress-induced cell death and accumulation of microsomes-associated and ubiquitinated αS species [38].

**Fig. 2:**
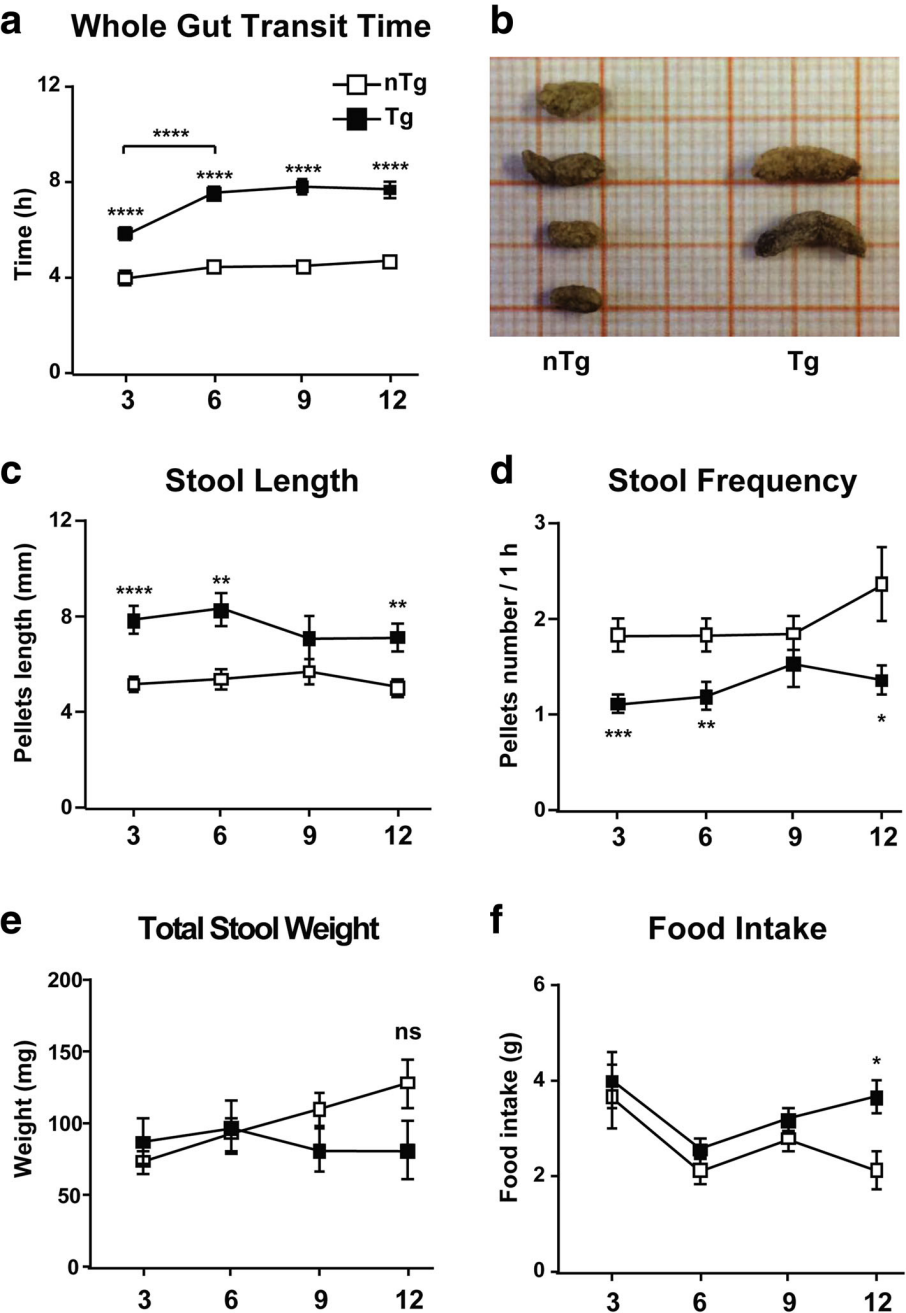
GI dysfunction in presymptomatic αS Tg mice is present starting from 3 months of age. GI functionality was evaluated through behavioral tests in presymptomatic 3, 6, 9 and 12 (if healthy) months old αS Tg mice and nTg littermates. Behavioral analysis showed significant constipation in young and adult Tg animals and nTg age-matched littermates. Each trial was performed 1 to 3 times per animal on non-consecutive days. Groups comprised of 20–30 mice with similar presence of females and males. **a** The Whole Gut Transit Time (WGTT) is increased in presymptomatic αS Tg mice versus controls already at 3 months and exacerbates with age, reaching an average 3 h 12 min delay in Tg animals by 6 months of age. The GI transit time was recorded as the time for a non-absorbable dye to travel through the GI tract. **b**, **c**, **d**, **e** Presymptomatic αS Tg mice excreted a reduced number of stools but with an increased length already at 3 months of age compared to controls, although the pellets total weight remained unchanged. **b** Representative image of stools collected in 1 h trial from a 6 month old αS Tg mouse and littermate. Notably pellets from young Tg mice are longer but less abundant. **c**, **d**, **e** Total pellets were collected in 1 h trail. Stools from Tg mice were consistently longer and less abundant although their total amount remained unchanged. **f** Food intake was measured in a 24 h trial. No significant difference between groups until 9 months of age in the amount of chow was found. Values on graphs are expressed as raw data and are given as the mean ± SEM (*n* = 20–30 per group). * *p* < 0.05; ** *p* < 0.01; *** *p* < 0.001, **** *p* < 0.0001, two-way ANOVA followed by Fischer’s LSD test

Once confirmed that αS Tg mice are free of pathology in the CNS at least until 9 months of age, we started to analyze GI functionality by evaluating the whole gut transit time (WGTT), that is, the time it takes for an edible dye to travel through the GI tract and to be excreted. At 3 months of age, the average transit time was 5.919 ± 0.2768 h for αS Tg mice and 4.117 ± 0.2751 h for nTg littermates, indicating that αS Tg mice had a transit delay of almost 2 h when compared to controls (*p* < 0.0001). This difference increased over time, reaching a plateau at 6 months of age [the average transit time was 7.680 ± 0.2419 h for αS Tg mice and 4.555 ± 0.1745 h for nTg mice, (*p* < 0.0001)], with a delay of more than 3 h compared to nTg littermates that was maintained later with aging (Fig. 2a). Since such a slowed transit time can affect the amount of pellets expelled, we measured the total stool weight and water content in young and adult Tg mice. To our surprise, stool weight recorded in a 1 h period (Fig. 2e) and related water content (Additional file 1, Figure S1b) remained unchanged between the two groups of mice at all the time points considered. Likewise, stool consistency and growth, measured as body weight and feeding behavior, were comparable between Tgs and nTgs up to 9 months of age, while no food malabsorption, such as in the event of episodes of vomit or diarrhoea, or hyperglycemia, was observed (Additional file 1: Figure S1c, d, e, f), suggesting that the marked delay in transit time was more likely to be related to a GI dysmotility rather than food digestion. In agreement with this, when the frequency of stool output by each single animal was measured in a 1 h trial, αS Tgs at 3 months of age showed an already 40% reduction in number of stools (2.243 ± 0.2210 for αS Tgs versus 3.670 ± 0.3458 for nTgs, *p* value = 0.00048) and a correlated 1.4-fold increase in stool length [7.864 ± 0.477 for αS Tgs versus 5.599 ± 0.242 for nTgs, (*p* < 0.0001)] (Fig. 2b, c, d). This difference was maintained in presymptomatic 6 month old mice but not at 9 months, suggesting that at 9 months other pathological processes might have affected directly or indirectly GI functionality in Tg animals and some mice might have been already committed to develop shortly αS-driven neuronal pathology in the brain. In line with this hypothesis, from 9 months of age αS Tgs showed a slight reduction in body weight and food intake consistent with changes in feeding behavior typical of motor symptoms onset (Fig. 2e, f). Thus young Tg mice show signs of constipation and GI abnormalities in presymptomatic conditions that greatly anticipate the appearance of the αS pathology in the CNS. In addition, the drastic increase in WGTT in young αS Tg mice did not translate into a decrease of the total amount of stools but rather affected the number and length of single pellets excreted, suggesting a defect in bowel propulsive motility since an early age.

### Presymptomatic αS Tg mice show a reduced electrically evoked motor response of the colonic muscle layers since 3 months of age

**Fig. 3:**
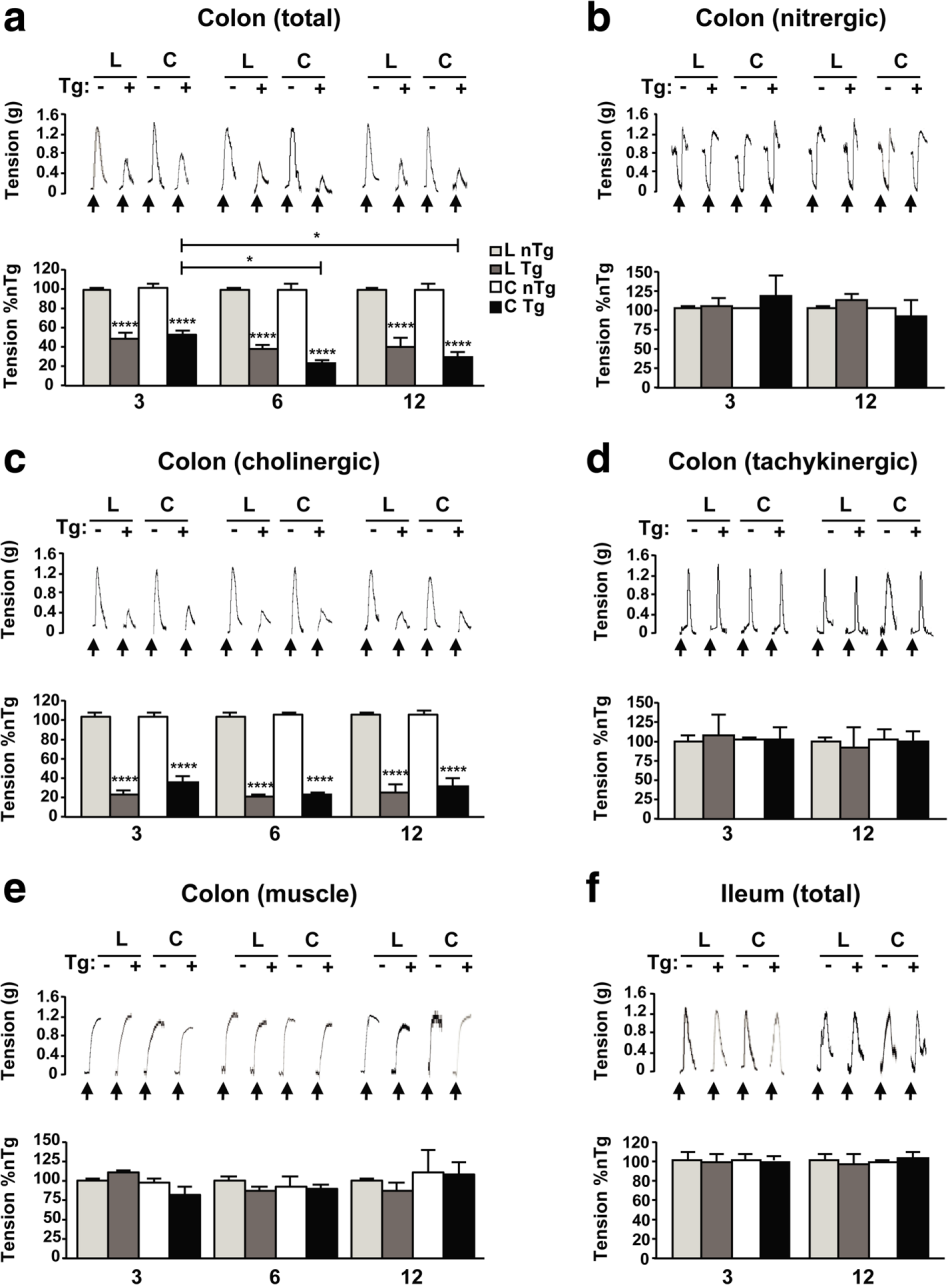
Colonic contractile activity is impaired in presymptomatic αS Tg mice from 3 months of age. Electrical stimulation (ES) of intestinal tissues shows a reduced response for the colon longitudinal (L) and circular (C) smooth muscles in Tg mice already at 3 months but not for the ileum. The reduced contraction was mainly due to a decreased cholinergic transmission and exacerbated with age. Recordings were performed in αS Tg mice and nTg littermate controls in colon (**a**, **b**, **c**, **d**, **e**) and ileum (**f**). **a**, **f** Effects of ES (↑ = ES, 10 Hz) on the contractile activity of colonic (**a**) or ileal (**f**) preparations maintained in standard Krebs solution in order to register the overall response. **b** Effect of selective ES (↑ = ES, 10 Hz) of the inhibitory nitrergic pathway on the activity of colonic longitudinal and circular muscle in order to record nitrergic-induced response. Colonic preparations were maintained in Krebs solution containing guanethidine, L-732,138, GR159897, SB218795 and atropine. **c** Effect of selective ES (↑ = ES, 10 Hz) of the excitatory cholinergic system on the activity of colonic muscle in order to record cholinergic-induced contractions. Colonic sections were maintained in Krebs solution containing L-NAME, guanethidine, L-732,138, GR159897, and SB218795. **d** Effect of selective ES (↑ = ES, 10 Hz) of the excitatory tachykinergic pathway on the activity of colonic muscle to record NK1-mediated tachykinergic contractions. Colonic preparations were maintained in Krebs solution containing L-NAME, guanethidine, GR159897, SB218795, atropine. **e** Effects of carbachol (↑ = Cch; 10 μM) stimulation on the activity of colonic preparations in order to record myogenic response in absence of neurogenic stimulation. Colonic preparations were maintained in Krebs solution containing tetrodotoxin. Tracings in the inset on the top of each panel display the contractile responses to ES, as raw data. Values on graphs are expressed as % relative to controls and are given as the mean ± SEM (*n* = 8 per group). * *p* < 0.05; **** *p* < 0.0001, two-way ANOVA followed by Fischer’s LSD test

Since constipation is correlated with a motility deficit of the intestine, electrophysiological recordings of longitudinal and circular muscle contractility of the intestinal wall were performed in both young and adult αS Tg mice. Electrical stimulation of the colon from αS Tg mice showed a clear reduction in the magnitude of motor responses of both muscle layers that was already present in Tg mice at 3 months of age compared to controls. In particular, the circular muscle seemed more affected since its response to stimulation was increasingly reduced with aging in αS Tgs (Fig. 3a). By contrast, in control animals, electrically evoked contractions showed consistently similar patterns of magnitude at all ages analyzed. To establish whether the reduced motor response in the colon of αS Tg animals was due to an impairment of a specific system within the ENS, we recorded the contractile response after selective stimulation of the nitrergic, cholinergic or tachykinergic system, pharmacologically isolated using specific inhibitors (Fig. 3b, c, d). Remarkably, only the excitatory cholinergic component was consistently impaired at all ages analyzed, in Tgs compared to age-matched littermates, as shown by the decrease of the electrically evoked motor response, while the other systems appeared not to be affected. This deficit in cholinergic transmission indicates that the increase in WGTT with concomitant alteration in stool formation is mainly due to a decrease in colon muscle contraction, rather than a dysfunction in muscle relaxation, which is mainly modulated in mice by the nitrergic system [39]. Furthermore, in order to understand whether this deficit was only neurogenic or also due to muscle abnormalities, we evoked colonic contractions by direct pharmacological activation of muscarinic receptors located on smooth muscle cells, using tetrodotoxin and carbachol (Fig. 3e). Interestingly, in this case the motor response was comparable between animal groups at all ages analyzed, indicating that the observed colonic dysmotility of Tg animals was mainly related to a deficit in the neural component. In addition, since the WGTT is a measure of the transit time for the whole GI tract, electrical stimulation of ileum preparations from presymptomatic mice at 3 and 12 months old was performed (Fig. 3f). To our surprise, motor response in the ileum was comparable between groups of mice (young and adult), indicating the deficit in contraction seen in young Tg mice was specific for the colon. Thus, the reduced contractility of the colon, due to an altered cholinergic transmission, in presymptomatic mice contributes to the abnormal formation of stools and their delayed GI transit time.

### Accumulation of αS soluble and insoluble HMW species in the colon increases over time in presymptomatic αS Tg mice

**Fig. 4:**
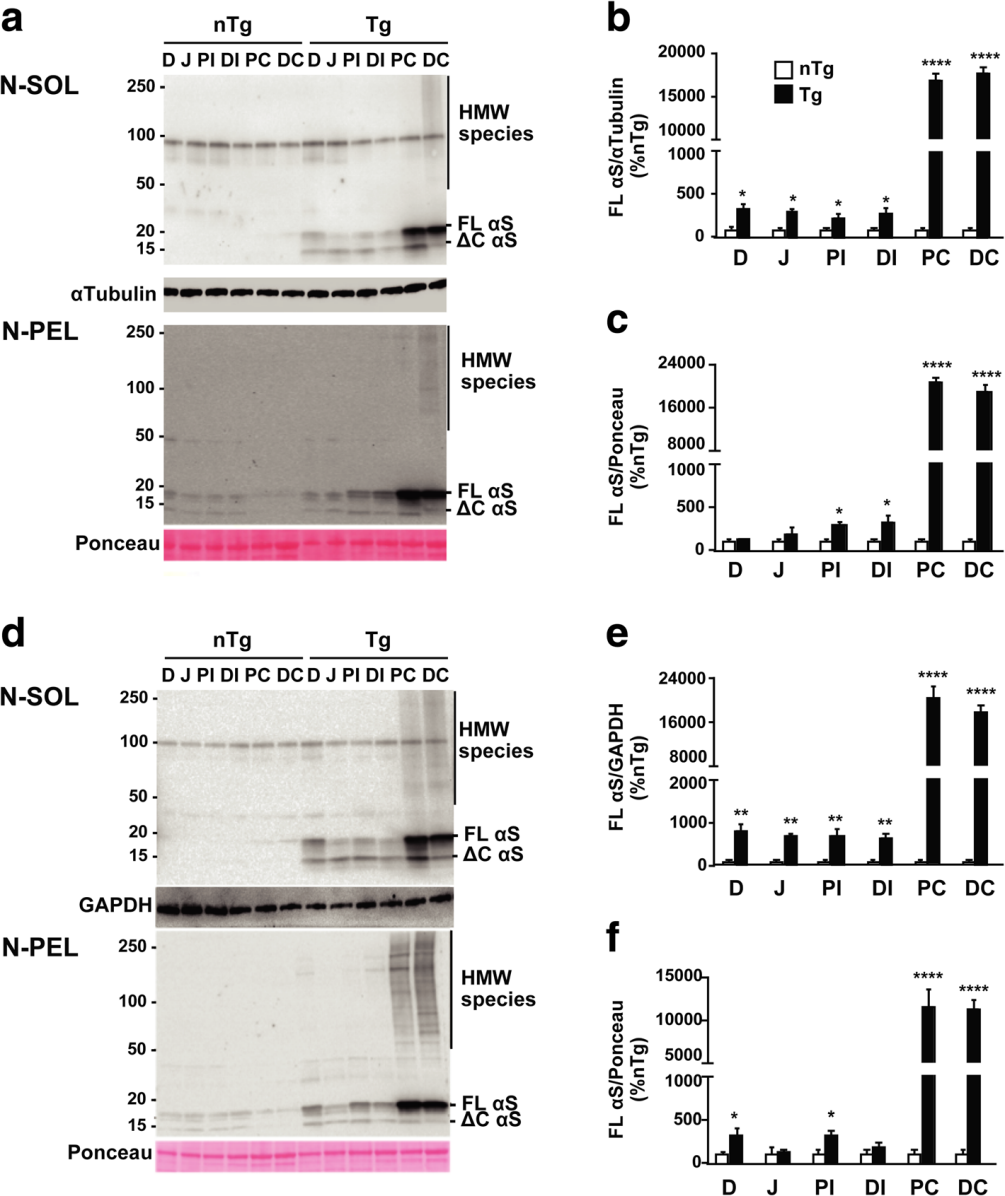
Intestinal αS distribution in presymptomatic and diseased αS Tg mice. Distribution of αS transgene expression was assessed in young presymptomatic (3 months old) and diseased Tg mice. Fresh mouse intestinal tract was divided in six segments corresponding to anatomical distinct areas: duodenum (D), jejunum (J), proximal ileum (PI), distal ileum (DI), proximal colon (PC), distal colon (DC). NP-40 soluble (N-SOL) and insoluble (N-PEL) fractions were loaded on a SDS-Page and blotted for Syn-1 and α-tubulin or GAPDH antibodies. αS expression was more abundant in the colon of both 3 months old presymptomatic (**a**) or sick αSTg (**d**) mice compared to the small intestine (*p* < 0.0001) or nTg age-matched littermates lysates, although it was present at minimal level in all the fractions examined in Tg mice. Notably insoluble and more surprisingly soluble fraction contained high molecular weight (HMW) αS species. FL αS, full length αS, ΔC αS, truncated αS. **b**, **c**, **e**, **f** Relative density of αS monomer in soluble (**b**, **e**) or insoluble (**c**, **f**) fractions. Values on graphs are expressed as % relative to nTg and are given as the mean ± SEM (*n* = 3–4 per group). * *p* < 0.05; **** *p* < 0.0001, two-way ANOVA followed by Fischer’s LSD test

**Fig. 5:**
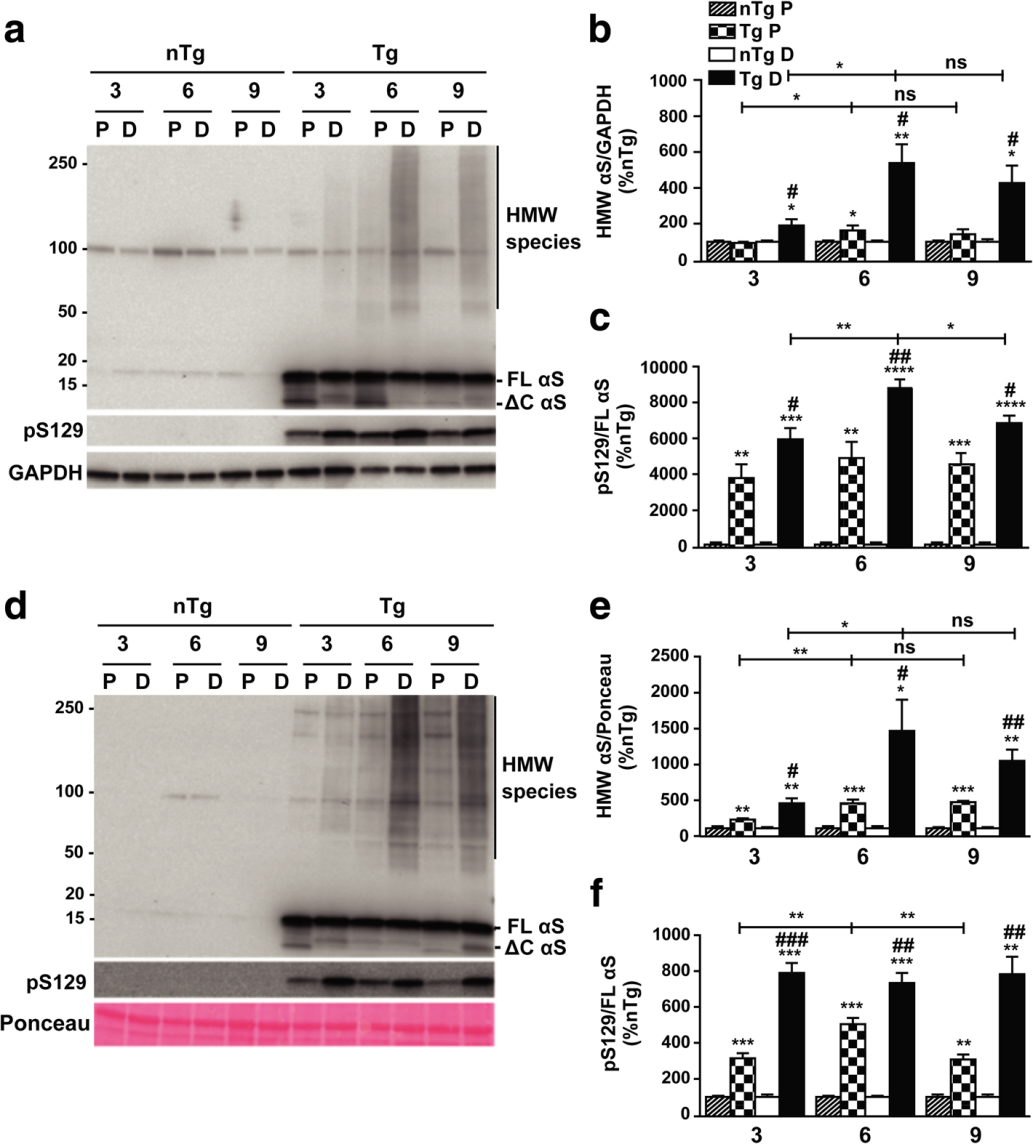
αS soluble and insoluble HMW species in the colon increase with age in presymptomatic mice. Time course analysis of soluble and insoluble fractions obtained from the proximal (P) and distal (D) colon of presymptomatic Tg mice at different age shows an increase in the accumulation of αS HMW species. NP-40 soluble (**a**) and insoluble (**d**) lysates of proximal and distal colon were isolated from presymptomatic Tg mice and nTg littermates at different age, run on a SDS-Page and blotted with Syn-1, pSer129-αS and GAPDH antibodies. Soluble and insoluble phospho-HMW αS species were already abundantly present in mice at 3 months of age and their level exacerbated with age, reaching a plateau at 6 months. The distal colon appears to be the intestinal region with the highest presence of αS oligomers and aggregates. **b**, **c**, **e**, **f** Graphs showing relative density of HMW αS (**b**, **e**) or phospho-αS (**c**, **f**) in all the fractions analyzed. Values on graphs are expressed as % relative to nTg and are given as the mean ± SEM (*n* = 3–4 per group). * *p* < 0.05; ** *p* < 0.01; *** *p* < 0.001; **** *p *< 0.0001, # *p* < 0.05; ## *p* < 0.01; ### p < 0.001; #### p < 0.0001, where * refers to comparisons between Tgs and their nTg age-matched controls, while # refers to comparisons between distal and proximal colon of Tgs; two-way ANOVA followed by Fischer’s LSD test

In order to investigate whether expression of αS could be related to the GI deficits that we observed we performed immunoblot analysis on the intestine of young and diseased animals to check for peripheral distribution of αS. The whole length of the intestine was examined from the pylorus to the rectum, dividing it into 6 segments corresponding to duodenum, jejunum, proximal and distal ileum, proximal and distal colon. Western blot assay showed that the αS monomer was present at low level in all the fractions analyzed with the exception of the colon where αS transgene expression reached a ~ 60-fold increase, compared to the small intestine, in presymptomatic 3 months Tg animals (Fig. 4). Very interestingly, when the NP-40 insoluble fraction was run, 3 months old presymptomatic αS Tg mice already showed accumulation in the colon (mainly in the distal segment) of aggregated, high molecular weight (HMW) species of αS. In diseased mice, after CNS neuronal degeneration onset, αS distribution was similar to that found in 3 months old mice, although the level of HMW αS was higher, suggesting a possible increase of αS inclusions formation with age. Indeed, when the proximal and distal colon from different ages was specifically analyzed for αS aggregates, we found an increased accumulation of insoluble and aggregated species of αS over time for both segments, although the distal colon appeared more affected, that peaked at 6 months and remained stable at later time points (Fig. 5d, e, f) whereas the αS monomer level did not change (Additional file 2: Figure S2). Notably, also the soluble fraction of the proximal and distal colon contained soluble HMW oligomers of αS, whose amount increased with aging and paralleled the trend seen for αS insoluble aggregates (Fig. 5a, b, c). In addition, phosphorylated αS at serine 129 was also detected in both insoluble and, more surprisingly, soluble fractions of the colon (Fig. 5c, f), a unique condition of the ENS, since phospho-αS species accumulate in the CNS of this line only after neurodegeneration onset and mainly in non-ionic detergent-insoluble pellet (Fig. 1) [13, 38, 40]. Thus, accumulation of phosphorylated αS inclusions in the colon of αS Tg mice largely precedes brain neuropathology, mimicking behavioral and physiology data. In addition, soluble αS is already phosphorylated at 3 months of age and forms stable, but still soluble, HMW oligomers, suggesting that pathobiology of αS may differ between the CNS and the ENS.

### Toxic αS in presymptomatic and diseased αS Tg mice is found in cholinergic and dopaminergic enteric neurons of the colon

**Fig. 6:**
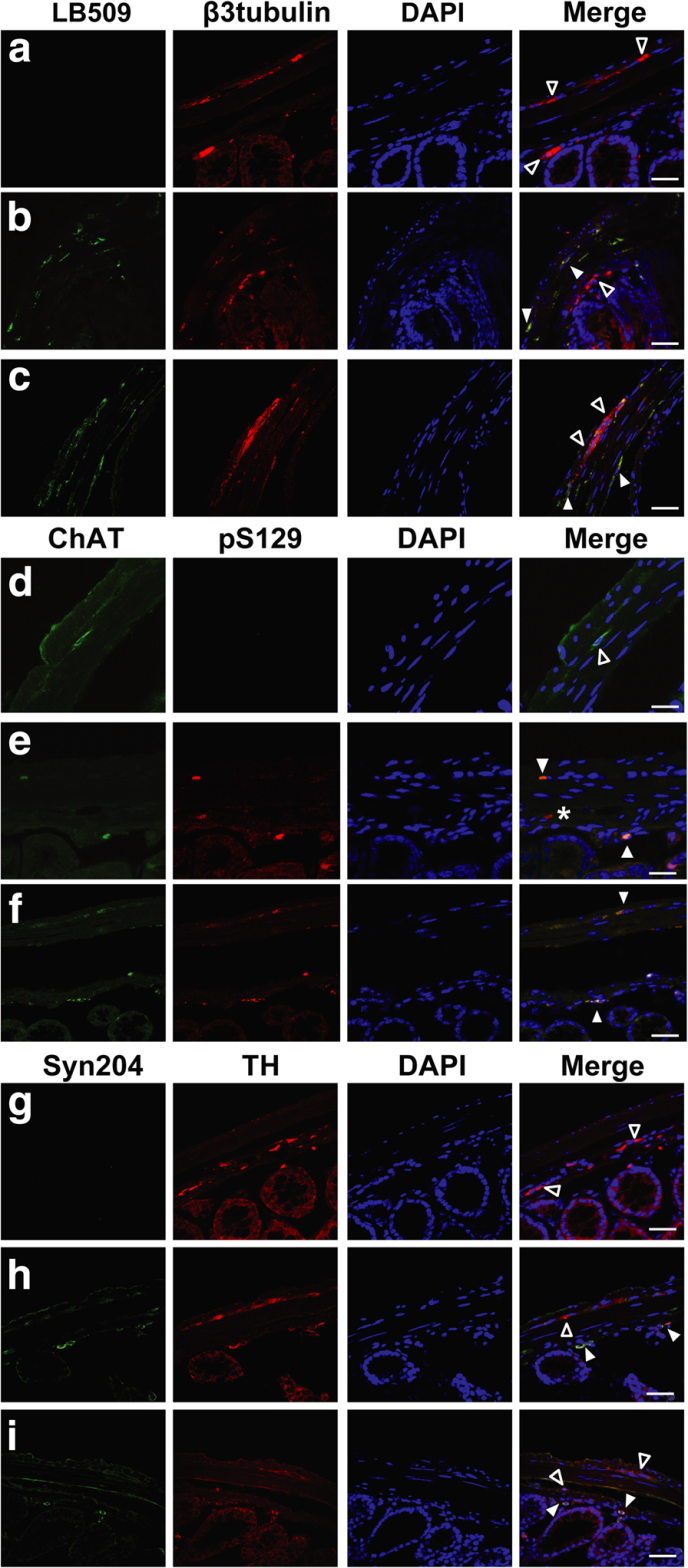
Accumulation of pathological αS in enteric neurons of presymptomatic and sick αS Tg mice. Immunocolocalization with neuronal markers shows accumulation of αS toxic species only in enteric neurons, including dopaminergic and cholinergic types, in both the submucosal and myenteric plexi in Tg mice but not in controls. Distal colon frozen sections from presymptomatic (3 months old) (**b**, **e**, **h**) and sick αS Tgs (**c**, **f**, **i**) and nTg littermates (**a**, **d**, **g**) were stained with LB509 and β3-tubulin (**a**, **b**, **c**); ChAT and pser129-αS (**d**, **e**, **f**); Syn204 and TH (**g**, **h**, **i**). Sections were counterstained with DAPI. Notably, within the same section and concurrently with double labeled neurons (full arrow heads), we found healthy neurons without accumulation of toxic αS (empty arrow heads) or neurons with accumulation of toxic αS that belonged to other neuronal populations beyond the cholinergic or dopaminergic system (*), suggesting that different enteric neurons may be distinctively susceptible to αS spreading. Images were acquired with Leica microscope SP2 system, objective 63x. Scale bars = 40 μm

In order to further investigate the distribution of pathological αS in the colon of Tg mice, we performed immunofluorescence analysis on frozen sections of the distal colon in presymptomatic, 3 month old and sick Tg mice. Colocalization with β3-tubulin, a pan-neuronal marker, showed that αS aggregates were present only in neurons, mainly in the soma, of both the myenteric and submucosal plexi of the ENS of Tg animals (Fig. 6a, b, c). Because of the deficit in cholinergic transmission observed in presymptomatic mice and since dopaminergic neurons are known to be particularly susceptible to αS toxicity [41], double immunostaining was carried out with specific neuronal-type markers to assess whether αS was specifically associated with these two neuronal populations. αS was indeed found in dopaminergic neurons (Fig. 6h, i) and in cholinergic neurons (Fig. 6e, f) of both enteric plexi. Interestingly, not all cholinergic and dopaminergic neurons stained for αS, which was concomitantly seen in other neuronal populations. This suggests a dynamic progression in the spreading and/or accumulation of toxic αS in the ENS. Thus accumulation of αS aggregates in colonic enteric neurons in both plexi is associated with onset of constipation and GI transit abnormalities that manifest in αS Tg mice in presymptomatic conditions without overt neurodegeneration in the CNS.

## Discussion

In this paper we show that the Prp A53T αS Tg mice, line G2–3, can be an extremely valuable model, and probably the only one, to the best of our knowledge, to study constipation as it occurs in PD prodromal phase, that is, in absence of overt motor abnormalities and CNS neurodegeneration. Together with typical motor symptoms upon which diagnosis is still mainly made, PD patients experience a variety of non-motor dysfunctions that affect both the CNS and the PNS [1]. Among these, GI dysfunction and specifically constipation represent an important feature of the prodromal phase of the pathology, due to the fact that GI-related symptoms can appear even decades prior the onset of the motor signs [3]. The relevance of studying constipation is also attributable to the fact that 80% of PD patients experience it and that after being diagnosed with constipation, there is almost a 3-fold increased risk of developing PD [5]. Nevertheless, whether there is a direct pathogenic correlation between GI dysfunction and PD still remains an open question, caused in part by the absence of appropriate animal models that recapitulate the chronic and progressive development of PD pathological stages. Indeed, all the αS Tg mouse lines analyzed for constipation so far showed GI deficit only concurrent with CNS neuropathology [30–32] while when considering the pharmacologically induced PD models only a small study, based on chronic injections of rotenone at low dosage in rats, showed mild GI abnormalities in absence of CNS αS pathology [42]. Nevertheless, because of the low amount of rotenone used for this purpose, brain pathology and motor symptoms never occurred in these mice. By contrast, in the G2–3 line constipation and GI dysfunction precede motor abnormalities and neurodegeneration in the CNS by at least 6 months. Indeed these mice from an early age (3 months old), in which the CNS is free of αS-driven neuropathology, manifest significant GI dysfunction, as shown by a robust increase in food transit time in the GI tract and simultaneous abnormalities in stool formation, which results in expulsion of elongated but less frequent pellets. Because of the similarities in stool consistency, food intake ration and body and stool weight between Tgs and age-matched nTg littermates, which allow us to exclude episodes of diarrhoea or chow malabsorption, the transit delay seen in young Tg mice could indicate a GI motility dysfunction in the process of propagation and expulsion of stools. Consistent with this, we found a decreased capacity of contraction of the longitudinal and circular muscle layers of the colon in young Tg mice that was mainly dictated by a reduced cholinergic transmission. Indeed, while the colon muscles in Tg mice can still contract normally when directly stimulated, it is the neuronal pathway supplying the underlying muscles that was found to be deficient. In addition, at 6 months of age, this GI abnormal behavior progressively worsens, reaching a delay in gut transit time of more than 3 h that was paralleled by a further decrease in colon contractility, still in absence of any CNS neurodegeneration.

Most of the neurotransmitters active in the CNS are also present in the ENS, with few differences, and contribute to the unrolling of all the diverse functions of the GI apparatus [43]. Specifically, the motor activity of the bowel is mainly regulated by the cholinergic and tachykinergic systems for contraction while the nitrergic system is the main regulator of gut muscle relaxation in mice [39, 44]. When considering only the recruitment of postganglionic cholinergic motor neurons, electrically evoked contractions resulted decreased in αS Tg compared to nTg mice in both muscle layers (Fig. 3b). On the other hand, when stimulating specifically the nitrergic or the tachykinergic system, the muscle responses were unchanged between the two groups, at all ages, confirming that GI delay in food transit was mainly ascribable to a deficit in cholinergic-evoked muscle contractions and not to an excess of the relaxant response. In line with this view, a more accurate immunohistochemistry analysis, found toxic αS accumulated in enteric neurons, including cholinergic and dopaminergic kinds, of both myenteric and submucosal plexi in presymptomatic and diseased animals. Notably, in the same section, not all the neurons stained for αS aggregates, indicating that accumulation of toxic αS is a progressive process where not all the neuronal populations present the same susceptibility to αS insults, especially in the gut where neuronal biodiversity is remarkable [43]. Aside from the renowned central dopaminergic dysfunction linked to the typical motor features of PD [41], other neuronal systems that can be related to both motor and non-motor symptoms, within and outside the CNS, can become affected in PD. In particular, the cholinergic system has been implicated in PD progression in relation to the occurrence of typical motor symptoms such as postural instability and gait disturbances [45], but also to non-motor symptoms such as dementia and cognitive deficits [46, 47], REM sleep behavior disorder [48], hyposmia [49], and possibly dysphagia [50], which may all manifest in preclinical PD. Interestingly, selective accumulation of LBs in cholinergic neurons in the nucleus basalis of Meynert, in the basal forebrain, has been described to occur concomitantly with dopaminergic neuronal loss in substantia nigra [9]. In addition, dopamine exerts a negligible control of intestinal motility at the level of the lower digestive tract in humans [44], strongly suggesting the implication of other neurotransmitters during the development of GI dysfunction in PD.

Moreover, in the present study we demonstrated that the bowel dysfunction shown by the behavioral and electrical recordings data in Tg mice is supported by a robust biochemical basis since immunoblot analysis of the whole intestinal tract revealed that young presymptomatic Tg mice predominantly express αS transgene only in the colon with consequent selective accumulation in this region of insoluble and phosphorylated HMW aggregates already at 3 months of age. Remarkably, while the distribution of αS transgene expression did not change with age, including after CNS pathology onset, the amount of insoluble aggregates increased over time, reaching a plateau at 6 months of age, temporally matching the increased delay in food transit and reduction of gut motility and providing at the same time a strong molecular basis to these behavioral and functional GI abnormalities. This strong correlation between presence of αS expression and LB-like αS aggregates in the colon and GI dysfunction is also confirmed by the absence of bowel dysmotility in the ileum of Tg mice, where αS expression is minimal. Thus GI αS inclusions, colonic neuronal deficit and GI dysmotility are tightly connected to αS overexpression in the gut in this mouse line and represent an early sign of αS-driven dysfunction, without CNS involvement. In PD patients, αS and LBs have been found in enteric neurons of both submucosal and myenteric plexi along the whole GI tract including the colon [8, 25]. An early investigation led by Beach and coworkers found a rostrocaudal gradient of accumulation of pser129-αS within the ENS with a higher incidence of LBs in the lower esophagus and submandibular gland and with less extent in the colon and rectum [7]. Although this observation has to be confirmed in a larger population, involvement of the vagal nerve, which directly innervates the stomach, the small intestine and the ascending colon, as the main dissemination route of toxic αS along the gut-brain axis, has been suggested, based also on the finding of LBs presence in the dorsal motor nucleus of the vagal nerve in prodromal PD [9]. However, the high incidence of aggregated αS in all the segments of the spinal cord suggests that other nerves might be implicated in αS propagation [25]. In addition, several studies have indicated LBs presence in human colon biopsies as a possible diagnostic method for preclinical PD [10, 24, 27]. Thus, while expression of human αS transgene in the gastric segment of the GI tract remains to be investigated in our mouse model, the early accumulation of toxic αS in the large intestine is sufficient in our mice to recapitulate distinctive features of GI dysfunction of human PD, making the G2–3 line an optimal paradigm to study constipation in premotor PD. In addition, and more surprisingly detergent-stable, soluble oligomers of αS were also found in presymptomatic 3 months old Tg mice and their accumulation increased over time, a striking difference with the CNS, where αS soluble oligomers are only found at low level in adult (> 9 months old) or diseased mice and mainly associated with the microsomal vesicles fraction [13, 40]. Furthermore, level of pser129-αS in the soluble fraction was also abundant, suggesting that αS pathobiology may differ between the brain and the gut, where modified, HMW αS remains soluble for a longer period of time, instead of being confined in insoluble LBs. While their toxicity remains to be investigated, increased solubility of αS toxic species may facilitate their spreading and tissue propagation.

## Conclusions

Constipation in the PrP human A53T αSTg mouse model, line G2–3, represents an early sign of αS pathology that manifests at least 6 months prior to motor symptoms and neuronal degeneration in the CNS. This net spatio-temporal separation of the two αS-driven pathologies makes the line G2–3 a unique tool to investigate the gut-brain connection in PD progression and to study GI dysfunction in prodromal PD.

## Additional files

Additional file 1: **Figure S1.** GI dysfunction in presymptomatic Tg mice. Feeding behavior, body weight and water content in stools are not affected in presymptomatic αS mice. Additional parameters related to GI functionality were evaluated through behavioral tests in presymptomatic αS Tg mice and nTg littermates. Each trial was performed 1 to 3 times per animal on non-consecutive days. Groups comprised of 20–30 mice for stool tests and body weight, or 10 mice for glycemic test, with similar presence of females and males. **a**, **b**) Pellets from each trial were weighted for total stool weight, then let dry o/n at 65 °C and weighted again to measure dry stool weight and water content. No difference in dry stool weight (**a**) nor water content (**b**) of the pellets was found between Tgs and controls at any time point. **c**, **d**) Glycemic levels were measured under fasted (**c**) and fed (**d**) conditions to assess whether feeding behavior was constant among animal groups. No significant differences between Tgs and age-matched controls were found indicating that GI dysfunction was not due to erratic food consumption. **e**) Body weight of the animals remained comparable between the two groups until 12 months of age, a time where surviving Tg mice may have been already committed to develop shortly the full motor phenotype. Values on graphs are expressed as raw data and are given as the mean ± SEM. * *p* < 0.05, two-way ANOVA followed by Fischer’s LSD test. (TIF 1075 kb)

Additional file 2: **Figure S2.** Distribution of αS monomer in the colon of presymptomatic Tg mice. Relative density of αS monomer in the proximal (P) and distal (D) colon of Tg mice and age-matched controls at various age shows that αS monomer level does not change with age. Quantitative analysis of immunoblots of soluble (**a**) and insoluble (**b**) fractions presented in Fig. 5. FL αS, full length αS. Values on graphs are expressed as % relative to nTg and are given as the mean ± SEM (*n* = 3/4 per group). **** *p* < 0.0001; two-way ANOVA followed by Fischer’s LSD test. (TIF 643 kb)

## Abbreviations

ENS: Enteric nervous system
ER: Endoplasmic reticulum
ES: Electrical stimulus
GI: Gastrointestinal
HMW: High molecular weight
LBs: Lewy bodies
PBS: Phosphate-buffered saline
PD: Parkinson’s disease
PNS: Peripheral nervous system
PrP: Prion protein
Tg: Transgenic
WGTT: Whole gut transit time
αS: Alpha-synuclein
