# Supplementary figures and images for: Constipation, deficit in colon contractions and alpha-synuclein inclusions within the colon precede motor abnormalities and neurodegeneration in the central nervous system in a mouse model of alpha-synucleinopathy
Source: Transl Neurodegener. 2019 Feb 6;8:5. doi: 10.1186/s40035-019-0146-z (PMC6364448; doi:10.1186/s40035-019-0146-z)

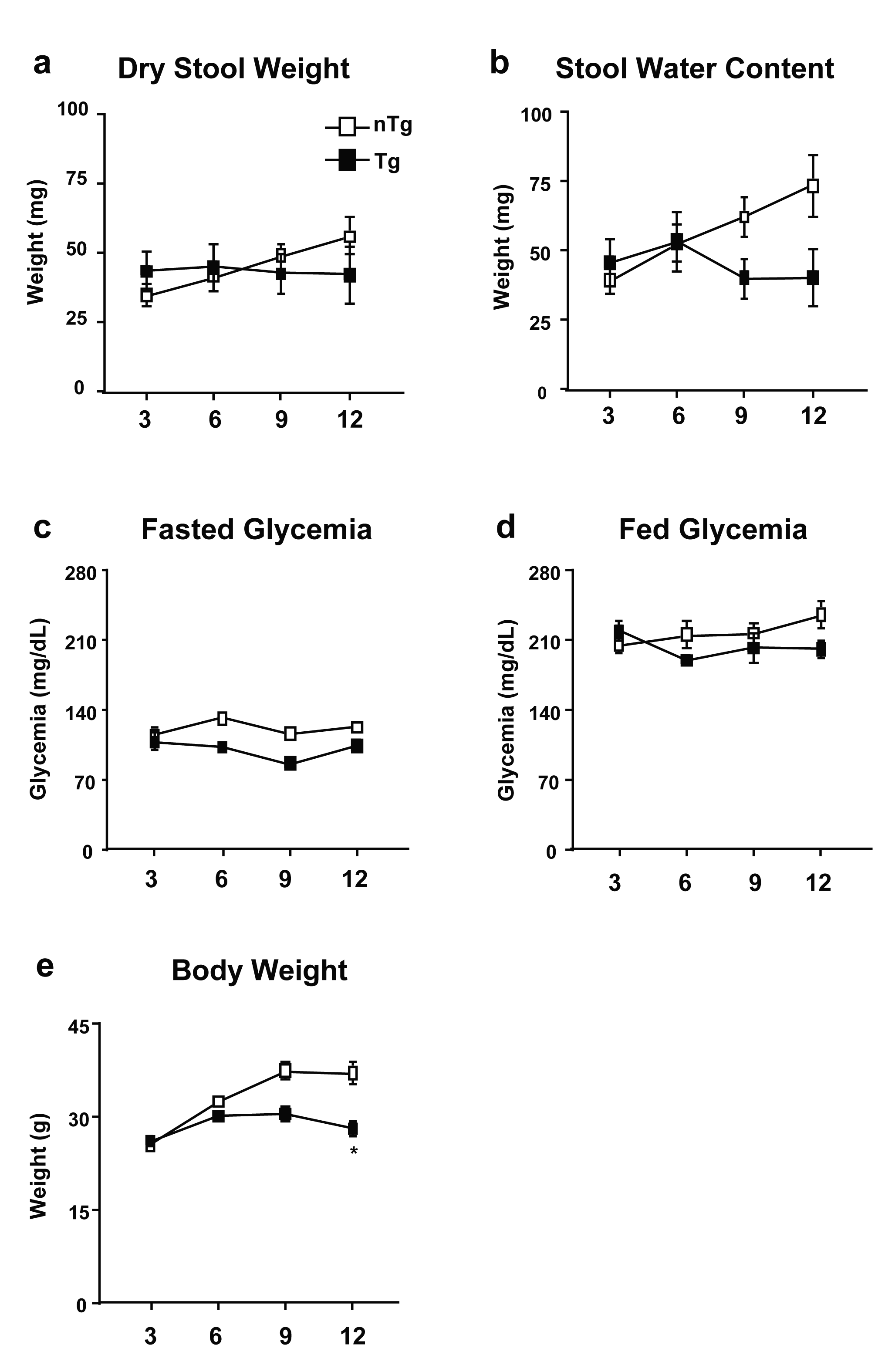

Supplement: Supplementary file 1 — Figure S1. GI dysfunction in presymptomatic Tg mice. Feeding behavior, body weight and water content in stools are not affected in presymptomatic αS mice. Additional parameters related to GI functionality were evaluated through behavioral tests in presymptomatic αS Tg mice and nTg littermates. Each trial was performed 1 to 3 times per animal on non-consecutive days. Groups comprised of 20–30 mice for stool tests and body weight, or 10 mice for glycemic test, with similar presence of females and males. a, b) Pellets from each trial were weighted for total stool weight, then let dry o/n at 65 °C and weighted again to measure dry stool weight and water content. No difference in dry stool weight (a) nor water content (b) of the pellets was found between Tgs and controls at any time point. c, d) Glycemic levels were measured under fasted (c) and fed (d) conditions to assess whether feeding behavior was constant among animal groups. No significant differences between Tgs and age-matched controls were found indicating that GI dysfunction was not due to erratic food consumption. e) Body weight of the animals remained comparable between the two groups until 12 months of age, a time where surviving Tg mice may have been already committed to develop shortly the full motor phenotype. Values on graphs are expressed as raw data and are given as the mean ± SEM. * p < 0.05, two-way ANOVA followed by Fischer’s LSD test. (TIF 1075 kb) [file 40035_2019_146_MOESM1_ESM.tif]

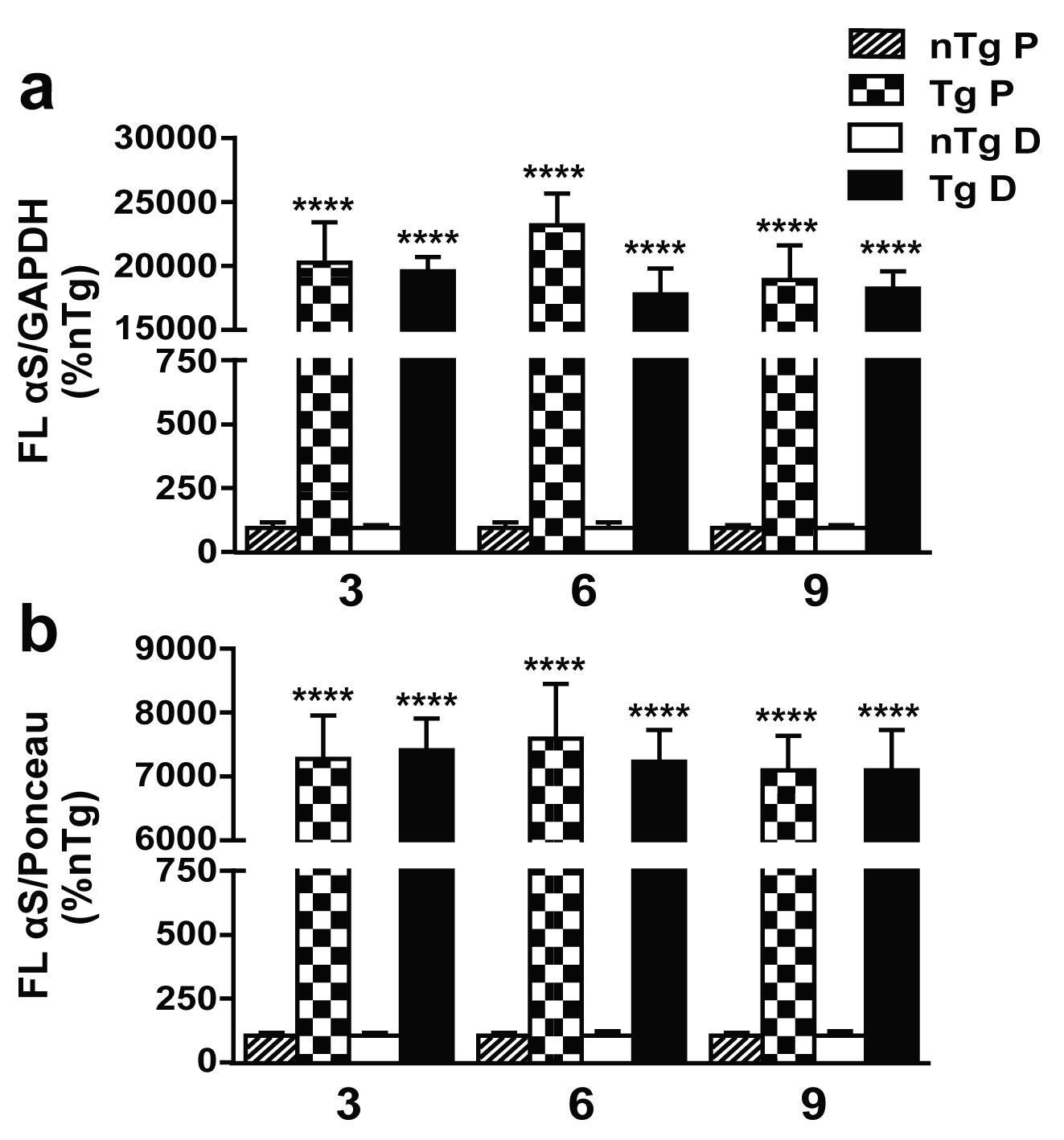

Supplement: Supplementary file 2 — Figure S2. Distribution of αS monomer in the colon of presymptomatic Tg mice. Relative density of αS monomer in the proximal (P) and distal (D) colon of Tg mice and age-matched controls at various age shows that αS monomer level does not change with age. Quantitative analysis of immunoblots of soluble (a) and insoluble (b) fractions presented in Fig. 5. FL αS, full length αS. Values on graphs are expressed as % relative to nTg and are given as the mean ± SEM (n = 3/4 per group). **** p < 0.0001; two-way ANOVA followed by Fischer’s LSD test. (TIF 643 kb) [file 40035_2019_146_MOESM2_ESM.tif]
